# Survival outcomes for HER2-low breast cancer: Danish national data

**DOI:** 10.2340/1651-226X.2024.41280

**Published:** 2024-11-14

**Authors:** Michael Sode, Kåre Nielsen, Maj-Britt Jensen, Tobias Berg, Ann Knoop, Bent Ejlertsen, Anne-Vibeke Lænkholm

**Affiliations:** a Department of Surgical Pathology, Zealand University Hospital, Roskilde, Denmark; b Danish Breast Cancer Group, DBCG, Rigshospitalet, Copenhagen University Hospital, Copenhagen, Denmark; c Department of Clinical Oncology, Rigshospitalet, Copenhagen University Hospital, Denmark, Copenhagen; d Department of Clinical Medicine, Faculty of Health and Medical Sciences, University of Copenhagen, Copenhagen, Denmark

**Keywords:** HER2-low breast cancer, survival analysis, HER2-low prognosis, HER2, DBCG

## Abstract

**Background and purpose:**

We investigated the prognosis of breast cancer (BC) with low expression of human epidermal growth factor receptor 2 (HER2), as previous studies have found varying impacts on survival of HER2-low BC compared with HER2 0 BC (HER2 IHC score of 0). HER2-low is defined as a score of 1+ or 2+ in an immunohistochemical (IHC) assay without *HER2* gene amplification.

**Materials and methods:**

Patients with HER2 0 or HER2-low BC from the national Danish Breast Cancer Group database were examined by multivariable survival analysis in a retrospective noninterventional investigation. Patients were grouped as either HER2 0 or HER2-low. The primary endpoint was time to recurrence (TR), and the secondary endpoints were overall survival (OS) and distant recurrence-free interval (DRFI).

**Results:**

41,610 patients were included (12,981 with HER2 0 BC and 28,629 with HER2-low BC). HER2-low BC was associated with a lower risk of recurrence (hazard ratio [HR]: 0.92, *p* = 0.03). Regarding secondary endpoints, HER2-low disease was linked to improved overall OS (HR: 0.94, *p* = 0.02). No statistically significant effect of HER2-low was found for DRFI, along with no differential effect of HER2-low according to estrogen receptor (ER) status.

**Interpretation:**

HER2-low BC was found to show an improved HR for OS and DRFI compared with HER2 0 BC; however, further studies are need to establish whether it represents a separate biological entity.

## Introduction

Breast cancer (BC) is the most common type of cancer in females worldwide and the second highest cancer-related cause of death. The disease is heterogeneous with both the histopathological and immunohistochemical classification having impact on the prognosis of BC patients [1, 2]. The molecular intrinsic subtypes are classified as Luminal A/B, HER2 enriched, and Basallike [2]. When applying surrogate immunohistochemistry for identification for the molecular intrinsic subtypes, the luminal subtypes are associated by the expression of estrogen receptor (ER), whereas the HER2-enriched molecular subtype mainly overexpresses the human epidermal growth factor receptor 2 (HER2) with important implications for the choice of targeted treatment and chemotherapy along with the prognosis [3]. Among patients with BC, treatment targeting HER2 was, until recently, limited to patients with HER2-positive disease, that is, patients with an immunohistochemical (IHC) HER2 score of 3+ and/or gene amplification [4–6]. However, the use of targeted anti-HER2 treatments has been extended to previously treated patients with HER2-low metastatic BC due to phase 3 results of the conjugated antibody Trastuzumab Deruxtecan (T-DXd) demonstrating a significant treatment effect on progression-free survival (PFS) and overall survival (OS) [7]. Additional trials within the HER2-low category are ongoing [8, 9]. The HER2-low category is defined by a HER2 score of 1+ or 2+ without detectable gene amplification and accounts for 40–60% of BC cases (with some studies reporting frequencies up to 70%), representing a large group of patients with potential benefit from new forms of HER2-targeted therapy in the metastatic or locally advanced setting [7, 10–13].

Conflicting results have been reported on the prognostic significance of HER2-low BC compared with HER2 0 with reports of both no effect [10–13] and better survival [14, 15].

In this retrospective population-based study, we investigated the clinical outcomes of HER2-low BC compared with HER2 0 in all Danish patients diagnosed from 2007 to 2019, who were treated with curative intent.

## Materials and methods

### Clinicopathological data

This retrospective, observational cohort study included all 50,714 female patients diagnosed with primary invasive HER2-negative BC in Denmark and treated with curative intent from January 1^st^ 2007 to December 31^st^ 2019 with follow-up until April 1^st^ 2022 (excluding stage IV BC). Since 1977, data on BC patients in Denmark have systematically been registered in the national Danish Breast Cancer Group (DBCG) database, including clinicopathological data and intended treatment at the time of diagnosis. In the DBCG database, follow-up is registered as either the first event or until a maximum of 10 years, whichever occurs first. The events recorded include death, contralateral BC, invasive recurrence (including the location(s) of recurrence), and other malignant disease. For a complete registration of the vital status, the DBCG database is linked to the Danish Civil Registration System [16]. From 2006, the database has been matched with the Danish Register for Pathology reports (Patobank) resulting in a close-to-complete coverage of patients with histopathologically verified BC.

From the DBCG database, the following categories/variables were extracted: Age at diagnosis, pathological features including HER2 IHC score, *HER2* gene copy number, and HER2/CEN17 ratio (if available), HER2 status (negative or positive), tumor size, histological subtype, histological grade (according to the Nottingham grading system), number of positive lymph nodes (LNs), ER status reported as a percentage of ER-positive tumor cells, and the Ki-67 index reported as a percentage of Ki-67-positive tumor cells; information about the intended treatment including surgical procedure, radiotherapy (RT), endocrine therapy (ET), and chemotherapy (CT); and site(s) of first recurrence. Management of BC in Denmark follows the national Danish guidelines from DBCG, in which the recommendations for HER2 testing from the American Society of Clinical Oncology/College of American Pathologists (ASCO/CAP) are employed [17–20]. In the case of HER2 IHC 2+ (equivocal), *HER2* ISH testing is recommended. The summarized guidelines followed by the Danish departments at the time for HER2 IHC and *HER2* ISH testing are displayed in Table 1.

**Table 1:** HER2 immunohistochemical (IHC) scoring (upper table) and in situ hybridization (ISH) interpretation (lower table) according to the recommendations in the 2007 [18], 2013 [20], and 2018 ASCO/CAP [20] guidelines.

HER2 score	ASCO/CAP 2007	ASCO/CAP 2013	ASCO/CAP 2018
**0 (negative)**	No staining	No staining or ≤10% of tumor cells with incomplete, faint, or barely perceptible staining.	No staining or ≤10% of tumor cells with incomplete, faint, or barely perceptible staining.
**1+ (negative)**	Weak, incomplete membrane staining in any proportion of tumor cells.	>10% of tumor cells with incomplete, faint membrane staining.	>10% of tumor cells with incomplete, faint membrane staining.
**2+ (equivocal)**	>10% of tumor cells with nonuniform or weak, circumferential staining, or intense membranous staining in ≤30% of tumor cells.	>10% of tumor cells with circumferential, incomplete, and/or weak to moderate membranous staining or ≤10% of tumor cells with circumferential, intense membranous staining.	>10% of tumor cells with complete, membranous staining.
**3+ (positive)**	>30% of tumor cells with uniform, intense membranous staining.	>10% of tumor cells with circumferential, intense membranous staining.	>10% of tumor cells with circumferential, intense membranous staining.
HER2 ISH	ASCO/CAP 2007	ASCO/CAP 2013	ASCO/CAP 2018
**Amplification**	*HER2* gene copy number >6 signals/tumor cell or *HER2/*CEP17 signal ratio >2.2	*HER2*/CEP17 signal ratio ≥2.0 or *HER2*/CEP17 signal ratio <2.0 with *HER2* gene copy number ≥6	*HER2*/CEP17 signal ratio ≥2.0 with *HER2* gene copy number ≥4, or *HER2*/CEP17 signal ratio <2.0 with *HER2* gene copy number ≥6
**Equivocal**	*HER2* gene copy number = 4–6 signals/tumor cells, or *HER2/*CEP17 signal ratio = 1.8–2.2	*HER2*/CEP17 signal ratio <2.0, with *HER2* gene copy number = 4-<6	*HER2*/CEP17 signal ratio <2.0, with *HER2* gene copy number = 4-<6
**Non-amplification**	*HER2* gene copy number <4 or *HER2/*CEP17 signal ratio <1.8	*HER2*/CEP17 signal ratio <2.0 with *HER2* gene copy number <4	*HER2*/CEP17 signal ratio <2.0 with *HER2* gene copy number <4, or *HER2*/CEP17 signal ratio ≥2.0 with *HER2* gene copy number <4

HER2: Human epidermal growth factor receptor 2; ASCO/CAP: American Society of Clinical Oncology/College of American Pathologists.

**Figure 1:**
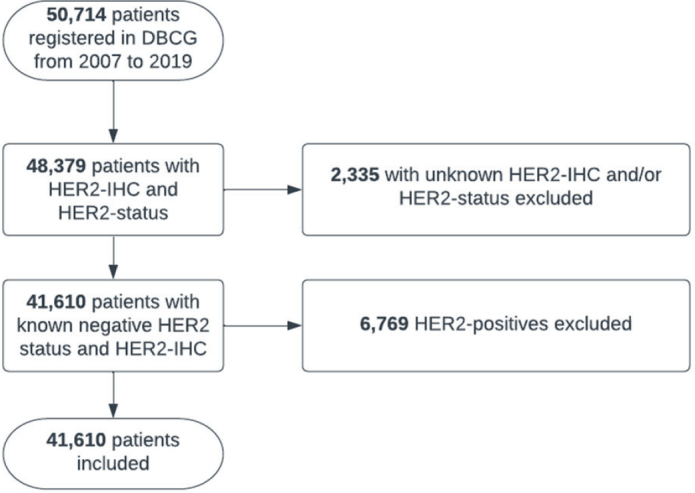
Flow chart of inclusion of patients in the cohort, which included all female patients diagnosed with invasive breast cancer in Denmark from 2007 to 2019 (excluding stage IV BC). A total of 41,610 patients were included.

During the study period, 50,714 female patients were diagnosed with BC, among whom 48,379 patients (95.4%) had recordings of both HER2 status (negative or positive) and HER2-IHC score (0, 1+, 2+, or 3+). Of these, 6,869 were excluded as HER2 positive (HER2 3+ and/or *HER2* amplification). The study cohort therefore consisted of 41,610 patients with HER2-negative BC (see flowchart in Figure 1).

### Primary and secondary survival outcome

The primary endpoint was time to recurrence (TR) defined as the time of diagnosis to recurrence. Contralateral BC, secondary carcinoma, and death as a first event were considered as competing risk events. Secondary endpoints included distant recurrence-free interval (DRFI) and OS. DRFI was defined as the time from diagnosis to distant invasive recurrence or death caused by BC. OS was defined as the time from diagnosis to death from any cause.

### Statistical analysis

Time of follow-up was calculated in terms of a Kaplan-Meier estimate of potential follow-up. TR and DRFI were estimated by cumulative incidence functions (CIF) due to the presence of competing risks. OS was calculated by Kaplan-Meier estimates.

The prognostic effect of HER2 low versus HER2 0 was quantified in univariable and multivariable analysis using the fine-gray proportional subdistribution hazard model for TR and DRFI due to the presence of competing risk events and the Cox proportional hazards regression model for OS. Statistical significance was assessed by the Gray test (TR and DRFI) and the log-rank test (OS).

The multivariable survival analysis included: Age at diagnosis (continuous), year of inclusion (cont.), tumor size (cont.), LN (cont. number of positive LNs), histological type and grade (cont. I, II, III), ER status (cont. 0–100%), local therapy (mastectomy vs. lumpectomy, +/- RT), and intention to treat (ITT) protocol (no treatment [low risk], endocrine therapy (ET), chemotherapy (CT), and combination [ET + CT]). Unknown values were analyzed in separate categories; sensitivity analysis was performed in a subset excluding the unknown values. Multivariable fractional polynomials were used to assess the functional form of parameters with a continuous measure. The proportional hazards assumption was tested using the Schoenfeld residuals and accommodated by including time-dependent variables with a cut-off point of 2.5 years in the models. For a sense of completion, the cut-off of 2.5 years is included in the multivariable survival analyses for TR and DRFI. To comply with the assumption of proportional hazards, a time-dependent component was also included for malignancy grade, ET, and RT (all DRFI, TR) at 2.5 years and for CT (DRFI, TR), grade, and ET (OS) at 5 years, and a continuously component for the year of diagnosis (TR, DRFI, OS) and ER level (OS, TR). An interaction model survival analysis was performed to assess effect modification between HER2 status and ER status grouped as 0%, 1–9%, and 10–100%. Unknowns were in separate models. The cut-off of 2.5 years was also included to ensure comparability with the previous results.

Descriptive statistics were applied to analyze the HER2 0 and HER2-low subgroups. A two-sided Pearson χ^2^ test was used to compare the distribution of the categorical variables between groups with unknowns separated into a separate category. Student’s T-test was used to compare normally distributed continuous variables and the Mann-Whitney test for other continuous variables.

All *p*-values are two sided, and the level of significance was set to 5%. Confidence intervals (CI) are reported as 95% CI.

Statistical analysis was performed using R, version 3.2.3 (R Project for Statistical Computing, (https://www.r-project.org/), SAS Enterprise Guide 7.15 HF7 (SAS Institute, Inc.), and STATASE17 (StataCorp).

## Results

### Clinicopathological characteristics

Among the 41,610 HER2-negative BCs, 12,981 (31%) were HER2 0, and 28,629 (69%) were HER2-low. Of the HER2-low patients, 22,322 (54%) were HER2 1+, but 6,307 (15%) were HER2 2+, respectively.

Table 2 displays demographical and pathological baseline characteristics for the patients. Median age at diagnosis was similar at 62 years for both HER2 0 and HER2-low. HER2-low status was more frequently associated with ER positivity than HER2 0 (92% vs. 82%, *p < 0.0001*). The mean tumor size was similar for HER2-0 and HER2-low (15 mm). HER2-low BC showed lower histological grade (*p < 0.0001*) and had more often positive LN status (*p < 0.0001*).

**Table 2:** Clinicopathological characteristics of the DBCG cohort (*n* = 41,610) and HER2 0 (*n* = 12,981) and HER2-low^a^ (*n* = 28,629). Clinical and pathologic information is from the DBCG register and includes females diagnosed with primary invasive BC from 2007 to 2019 in Denmark. All *p*-values were *P* < 0.0001.

Clinical and pathologic characteristics	DBCG cohort		HER2 0		HER2-low	
	No	%	No	%	No	%
**Total number of patients**	41,610	100	12,981	31	28,629	69
**Age, median (years), range^b^**	62 (20–100)		62 (23–98)		62 (20–100)	
**Menopause**						
**Premenopausal**	9,552	23	3,117	24	6,435	22
**Postmenopausal**	32,058	77	9,864	76	22,194	78
**Tumor size, mean (mm)^b^**	15		15		15	
**Malignancy grade**						
**Grade I**	12,131	29	3,481	27	8,650	30
**Grade II**	17,991	43	5,002	39	12,989	45
**Grade III**	7,300	18	2,883	22	4,417	15
**Not graded^c^**	3,832	9.2	1,487	11	2,345	8.2
**Unknown**	356	0.9	138	0.99	228	0.80
**Positive lymph nodes**						
**0**	24,953	60	7,946	61	17,007	59
**1–3**	11,079	27	3,221	25	7,858	27
**≥4**	3,628	8.7	1,074	8.3	2,565	9.0
**Unknown**	1,950	4.7	740	5.7	1,199	4.2
**ER status**						
**0**	4,391	11	2,324	18	2,067	7.2
**1–9%**	493	1.2	216	1.7	277	0.97
**10–99%**	9,967	24	2,807	22	7,160	25
**100%**	26,439	64	7,467	58	18,972	66
**Negative^d^**	261	0.63	142	1.1	119	0.42
**Positive^d^**	42	0.10	20	0.15	22	0.077
**Unknown**	17	0.041	5	0.039	12	0.042
**Subtype**						
**IDC**	32,730	79	9,888	76	22,842	80
**ILC**	5,035	12	1,599	12	3,436	12
**Other type of carcinoma**	3,820	9.2	1,478	11	2,342	8.2
**Not known**	25	0.060	16	0.12	9	0.031
**Intention to treat**						
**ET**	18,041	43,4%	5,018	38,7%	13,023	45,5%
**CT**	4,707	11,3%	2,492	19,2%	2,215	7,74%
**ET+CT**	13,555	32,6%	3,745	28,8%	9,810	34,3%
**None**	5,307	12,8%	1,726	13,3%	3,581	12,5%
**Radiotherapy**						
**No**	9,623	23	2,892	22	6,731	24
**Yes**	30,833	74	9,729	75	21,104	74
**Unknown**	1,154	2.8	360	2.8	794	2.8
**Surgery**						
**Mastectomy**	11,888	29	3,427	26	8,461	30
**Lumpectomy**	29,516	71	9,477	73	20,039	70
**No operation**	206	0.50	77	0.59	129	0.45

a HER2-low includes patients who scored HER2 1+/2+ without ISH HER2 gene amplification.

b Means and medians only include patients with known values.

c Not graded as the subtypes do not require it.

d Graded as ER-positive (10+%) or ER-negative (<10%).

IDC: Invasive Ductal Carcinoma; ILC: Invasive Lobular Carcinoma; ER: Estrogen Receptor; NACT: Neoadjuvant chemotherapy treatment; HER2: Human Epidermal growth factor Receptor 2; DBCG: Danish Breast Cancer Group; ET: endocrine therapy; CT: chemotherapy.

### Outcome in HER2-low and HER2 0 BC

**Figure 2:**
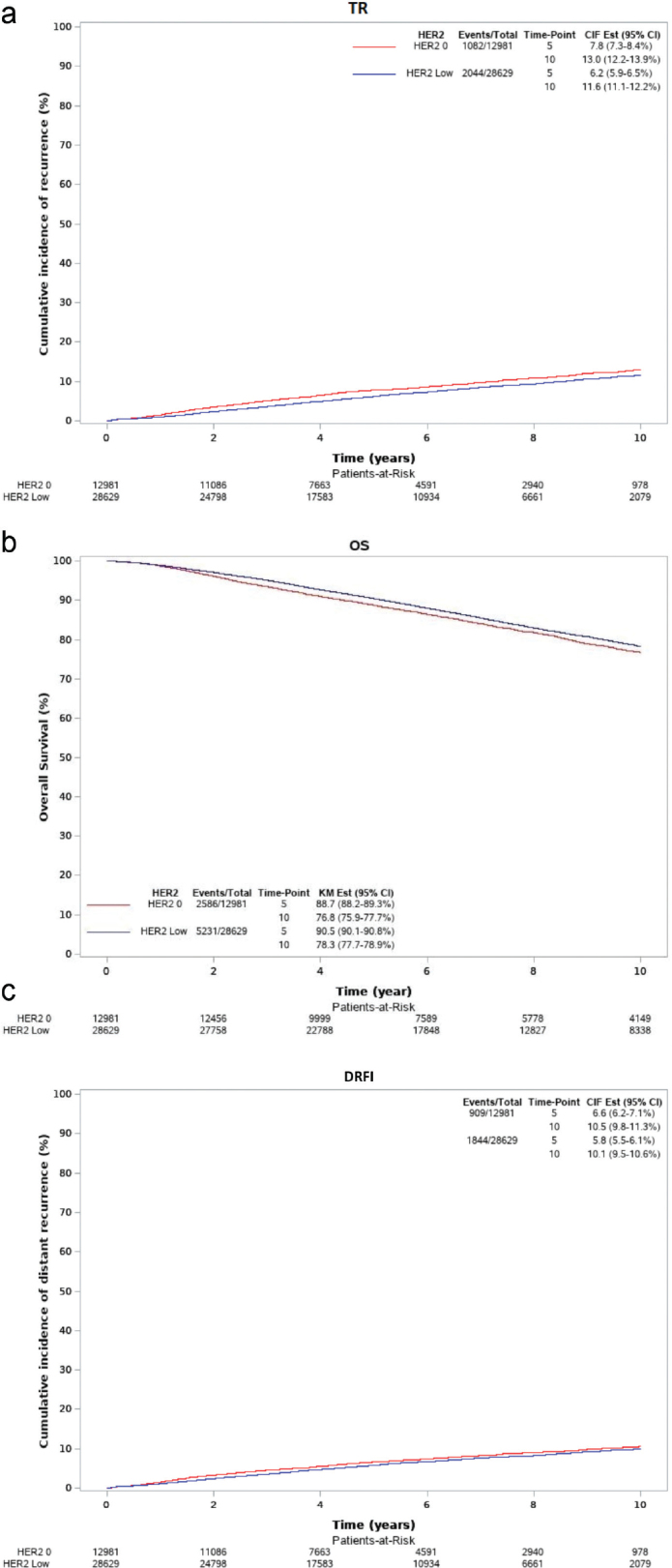
(a) Cumulative incidence function (CIF) curve for time to recurrence (TR). (b) Kaplan-Meier curves for overall survival (OS). (c) CIF curve for distant recurrence-free interval (DRFI).

Among all patients, the estimated median potential follow-up of TR and OS was 5.5 and 8.5 years, respectively. In the DBCG cohort, 3,126 patients had BC recurrence (TR), 7,817 patients were dead from any cause (OS), and 2,753 patients experienced distant recurrence or death from BC (DRFI). In Figure 2, the cumulative incidence for TR and DRFI and estimates of OS are displayed for patients with HER2 0 and HER2-low BC. The results from the univariable and multivariable survival analysis of HER2-low BC are presented in Table 3.

**Table 3:** Univariable and multivariable survival analyses for overall survival (OS) (Cox regression analyses), time to recurrence (TR), and distant recurrence-free interval (DRFI) (fine-gray subdistribution hazard model) for HER2-low with HER2 0 as a reference according to time after diagnosis.

	TR			OS			DRFI		
	Hazard ratio (95% CI)		*p*	Hazard ratio (95% CI)		*p*	Hazard ratio (95% CI)		*p*
**Univariable – HER2-low**									
**Overall**	0.84	0.78; 0.90	<0.0001	0.93	0.89; 0.97	0.002	0.90	0.83; 0.98	<0.0001
**0–2.5 years**	0.69	0.62; 0.77	<0.0001	0.73	0.66; 0.80	<0.0001	0.76	0.68; 0.85	<0.0001
**2.5- years**	0.97	0.88; 1.07		1.00	0.95; 1.06		1.07	0.96; 1.20	
		p_heterogeneity_	<0.0001		p_heterogeneity_	<0.0001		p_heterogeneity_	<0.0001
**Multivariable – HER2-low**									
**Overall**	0.92	0.85; 0.99	0.03	0.94	0.90; 0.99	0.02	0.96	0.89; 1.05	0.40
**0–2.5 years**	0.86	0.77; 0.97	0.03	0.85	0.77; 0.94	0.003	0.91	0.81; 1.03	0.32
**2.5- years**	0.96	0.87; 1.07		0.98	0.92; 1.03		1.02	0.90; 1.14	
		p_heterogeneity_	0.15		p_heterogeneity_	0.02		p_heterogeneity_	0.20

HER2: Human epidermal growth factor receptor 2; CI: confidence interval.

### Primary outcome

The 5-year TR of HER2-low and HER2 0 BC was 6.2% (CI: 5.9; 6.5) and 7.8% (CI: 7.3; 8.4), respectively. Correspondingly, the 10-year TR of HER2-low and HER2 0 BC was 11.6% (CI: 11.1; 12.2) and 13.0% (CI: 12.2; 13.9).

The overall HR of HER2-low TR compared with HER2 0 in the univariate survival analysis was 0.84 (CI: 0.78; 0.90, *p* < 0.0001). The test of heterogeneity was statistically significant (*p* < 0.0001) for TR 0–2.5 years and TR 2.5+ years: HR: 0.69 for TR 0–2.5 years (CI: 0.62; 0.77) and HR: 0.97 for TR 2.5+ years (CI: 0.88; 1.07), respectively.

The multivariate survival analysis showed that HER2-low TR had a lower overall HR of 0.92 (0.85; 0.99 *p* < 0.03). The test of heterogeneity according to time after diagnosis was not statistically significant (*p* = 0.15), but as previously stated, the divided intervals are included in the table.

### Secondary outcomes

For HER2-low BC patients, the 5-year OS was 90.5% (95% CI: 90.1; 90.8) and 10-year OS was 78.3% (95% CI: 77.7; 78.9). The corresponding figures for HER2 0 were 5-year OS of 88.7% (95% CI: 88.2; 89.3) and 10-year OS of 76.8% (95% CI: 75.9; 77.7). For DRFI, the 5-year figure for HER2-low and HER2 0 BC was 5.8% (95% CI: 5.5; 6.1) and 6.6% (95% CI: 6.2; 7.1), respectively, and the 10-year figure 10.1% (95% CI: 9.5; 10.6) and 10.5% (95% CI: 9.8; 11.3), respectively.

The overall HR of HER2-low versus HER2 0 for OS in the univariable analysis was 0.93 (95% CI: 0.89; 0.97, *p* = 0.03). The test of heterogeneity between the early and late effect was statistically significant at *p* < 0.0001. The same was seen for DRFI with an HR of 0.90 (95% CI: 0.83; 0.98, *p* < 0.0001) and a significant test of heterogeneity of *p* < 0.0001. The univariable survival analysis showed that HER2-low patients experienced a statistically significantly prolonged OS (hazard ratio [HR]: 0.73, 95% CI: 0.66; 0.80) and DRFI (HR: 0.76, 95% CI: 0.68; 0.85) for year 0–2.5 but not for year 2.5+ (for OS: HR: 1.00, 95% CI: 0.95; 1.06; for DRFI: HR: 1.07, 95% CI: 0.96; 1.20).

In the multivariable analysis, the HR for OS of HER2-low BC compared with HER2 0 BC was 0.94 (95% CI: 0.90; 0.99, *p* = 0.002) with a statistically significant test of heterogeneity at *p* = 0.02. The multivariable survival analysis showed that HER2-low patients had significantly longer OS in the initial 0–2.5 years (HR: 0.85, 95% CI: 0.77; 0.94) but not beyond 2.5 years (HR: 0.98, 95% CI: 0.92; 1.03). The overall HR for DRFI in the multivariable survival analysis was not statistically significant with an HR of 0.96 (95% CI: 0.89; 1.05, *p* = 0.40). For completion, the corresponding time-dependent HR for DRFI is also displayed in Table 3, but the test of heterogeneity was not statistically significant.

### Interaction between ER and HER2-low

The interaction analysis between HER2-low/HER2 0 BC and ER (0%, 1–9%, and 10–100%) for all three endpoints is presented in Table 4. For the primary outcome, TR, and the secondary outcome, DRFI, no statistically significant effect of HER2 status was observed based on the ER group. For the other secondary outcome, OS, the HER2-low group with ER 0% showed a lower HR of 0.87 compared with HER2 0 and ER 0% although this difference was not statistically significant. No significant heterogeneity of HER2 status according to the ER group was seen for any of the end-points.

**Table 4:** Multivariable survival analyses of HER2 status according to the ER expression for overall survival (OS), time to recurrence (TR), and distant recurrence-free interval (DRFI).

	TR			OS			DRFI		
	Hazard ratio (95% CI)		*p*	Hazard ratio (95% CI)		*p*	Hazard ratio (95% CI)		*p*
**HER2-low vs. HER2 0**			0.67			0.21			0.96
**ER 0%**	0.97	0.80; 1.16		0.87	0.78; 0.98		0.95	0.78; 1.14	
**ER 1–9%**	0.82	0.50; 1.33		1.14	0.79; 1.65		0.90	0.52; 1.57	
**ER 10–100%**	0.89	0.81; 0.97		0.96	0.91; 1.01		0.96	0.87; 1.06	
**0–2.5 years**			0.21			0.20			0.73
**ER 0%**	0.97	0.79; 1.19		0.80	0.68; 0.94		0.92	0.75; 1.13	
**ER 1–9%**	0.95	0.57; 1.60		1.21	0.78; 1.89		1.11	0.61; 2.03	
**ER 10–100%**	0.79	0.69; 0.90		0.87	0.77; 0.97		0.88	0.76; 1.02	
**2.5- yrs**			0.43			0.71			0.33
**ER 0%**	0.96	0.72; 1.27		0.93	0.81; 1.08		1.02	0.73; 1.43	
**ER 1–9%**	0.59	0.29; 1.21		1.08	0.70; 1.65		0.55	0.24; 1.24	
**ER 10–100%**	0.95	0.86; 1.06		0.99	0.93; 1.05		1.02	0.90; 1.15	
		p_heterogeneity_	0.22		p_heterogeneity_	0.49		p_heterogeneity_	0.38

ER: Estrogen receptor; HER2: Human epidermal growth factor receptor 2; CI: confidence interval.

## Discussion

In the DBCG cohort, HER2-low BC was associated with lower histological grade, higher ER-positivity rate, and more frequent LN involvement, compared with HER2 0 BC. This is consistent with other studies regarding histological grade, ER expression, and LN positivity rate [14, 21, 22]. Of particular note, a large investigation of 1,136,016 patients with invasive BC from the US National Cancer Database found that HER2-low BC was associated with a higher proportion of ER-positive BC and higher LN staging [23].

Preclinical experiments have provided evidence of a bidirectional ‘crosstalk’ molecular pathway between ER and HER2 in ER-positive/HER2-positive cancers. Both in vitro and in vivo studies have shown that treatments targeting a single pathway can lead to the upregulation of the corresponding pathway, resulting in acquired treatment resistance and potential ‘escape pathways’ from the targeted therapy [24, 25]. Clinical trials have supported this hypothesis although some divergent results have been attributed to variations in trial designs and methodologies [24].

Traditionally, *ER* and *HER2* RNA levels have been viewed as inversely related [24, 26]. However, a 2012 study found a positive correlation of HER2 RNA levels between HER2 0 and HER2 1 within HER2-negative BC [26]. This bimodal distribution of ER expression based on HER2 levels raises intriguing questions for further investigation into the molecular relationship at lower levels of HER2 expression, especially in light of new targeted treatments [24]. Nevertheless, our results did not demonstrate a differential effect of ER status on survival outcomes, and additional experiments and studies are needed to clarify the molecular, genetic, and proteomic basis for these differences.

The multivariable survival analysis showed that HER2-low status was associated with a statistically significant lower HR for the primary clinical outcome TR compared with HER2 0 BC. As regard the secondary clinical outcomes, HER2-low status was an independent prognostic factor for OS, both overall from 0 to 2.5 years with a statistically significant test of heterogeneity (*p* = 0.02) at year 2.5.

Retrospective studies have reported variable prognostic significance of HER2-low BC compared with HER2 0 BC, with findings ranging from no effect to a modest positive effect [11, 14, 15, 22]. Notably, the aforementioned study from the US National Cancer Database indicated a marginally improved 5-year OS for HER2-low BC, aligning with our results [23]. A meta-analysis of published studies found that HER2-low status was associated with significantly improved OS (HR: 0.84, 95% CI: 0.77; 0.92, *p* < *0.001*) and disease-free survival (DSF) (HR: 0.86, 95% CI: 0.79; 0.92, *p* < *0.001*) in early-stage settings but not in metastatic cases [8]. However, an expert consensus statement from the European Society for Medical Oncology concluded that there is insufficient evidence to support a prognostic value of HER2 expression within HER2-negative BC, citing inconsistencies in the results and small sample sizes of most studies [9].

A portion of the observed variance can be attributed to interobserver and interlaboratory differences, particularly within the HER2 0 and HER2 1+ categories [27]. One significant challenge of this retrospective study design is the variability in HER2 detection methods across laboratories and observers in current clinical practice. Moreover, existing HER2 IHC testing guidelines primarily focus on differentiating between HER2 overexpression and normal expression, rather than accurately assessing lower levels of HER2 expression. In the DBCG register, 56% of BC cases were classified as HER2 low, while other studies have reported this figure to be around 40–50% of clinical HER2-negative BC [12]. A study from the author group found high inter-laboratory variation of HER2-low BC in our cohort ranging from 49.3 to 65.6% among Danish laboratories [28]. This variability may help explain the discrepancies in prognostic outcomes for HER2-low BC observed in various studies and underscores the urgent need for standardization and a greater focus on HER2-low cases, potentially including the use of alternative assay [29]. Other reasons for the differing results in the survival studies of HER2-low BC could be the use of different endpoints and different follow-up intervals and variance in the makeup of the cohorts. Additionally, differences in study design, such as the choice of endpoints, follow-up intervals, and the composition of patient cohorts, could further account for the varying results reported in survival studies of HER2-low BC.

In our study, HER2 status was tested on the primary tumor, and the status of HER2 is therefore not immediately available for any metastasis, because this is not routinely tested in clinical practice. A study looking at paired samples of primary and recurrent BC found a relatively low HER2 discordance rate of 25.9% between primary and recurrent HER2 status when looking at primary HER2-low BC with similar results in other studies [30]. A study of 98 patients found a discordance rate of 27.6 % between the HER2-low BC primary tumor and the metastasis with the majority of 81.5 % being downgraded to HER2-0 [30]. They found no significant statistical differences between HER2 0 and HER2-low BC concerning OS, DFS, and PFS in metastasis with different HER2-status from the primary site. Additionally, they found that HER2 discordance increased with the tumor size and was higher in surgical specimens compared with biopsies, which they attributed this partly to intratumor heterogeneity of HER2 expression along with different biological subpopulations of the tumor cells [30]. With the advent of new therapeutic agents for HER2, testing of metastatic lesions is mandatory in Denmark [31].

No interaction was found between ER status and HER2-low in our results. The relationship between ER and HER2 at low levels of expression, particularly regarding its impact on survival, has not been well studied compared with ER and HER2-positive BC. For instance, a study examining HER2-low in HER2-negative/ER-positive BC found no interaction between HER2-low and ER expression (measured as a continuous percentage) in relation to PFS [32]. Denkert et al. reported that HER2-low disease-free survival was lower with ER-negative BC, but not with ER-positive BC [15]. Coriano et al. observed that there was a longer OS and higher overall pCR in the HER2-low/ER-positive subgroup [33]. Other studies looking at ER-negative and -positive BC (defined as ER ≥ 10%) have found no difference between HER2-low and HER2 0 [34].

A strength of this study is that the dataset is collected on a national basis with a close-to-complete coverage and with widespread standardization of the guidelines for management, diagnostic workup, and pathological examination. One limitation of this study, which is comparable with other studies investigating the prognosis of HER2-low BC, is the retrospective design and the lack of central review along with the use of ITT protocols. The ASCO/CAP guidelines were also updated during the study period (see Table 1), which changed the HER2 scoring scale, possibly accounting for some variance in the HER2-low category.

## Conclusion

This survival study represents one of the largest retrospective investigations of the prognosis of HER2-low BC. HER2-low status was associated with a longer TR and higher OS compared with HER2 0. No interaction between ER and HER2 status was found. Further prospective studies are needed to establish whether HER2-low BC represents a separate subgroup.

## Statements & Declarations

### Data availability statement

The data in this study are administered by The Danish Clinical Quality Program (https://www.rkkp.dk) and can be acquired by applying to this program.
